# Leiomyosarcoma Originating From Axilla in Neurofibromatosis Type 1: A Rare Occurrence

**DOI:** 10.7759/cureus.39007

**Published:** 2023-05-14

**Authors:** Assam Ullah, Salman Khan, Muhammad Irfan, Imad Majeed, Imadullah Khan, Hameed Haidar Khan

**Affiliations:** 1 Department of Medicine, Khyber Teaching Hospital, Peshawar, PAK; 2 Department of Cardiology, Hayatabad Medical Complex, Peshawar, PAK

**Keywords:** sarcoma soft tissue, von recklinghausen syndrome, soft tissue tumor, leiomyosarcoma, neurofibromatosis type 1

## Abstract

Neurofibromatosis type 1 (NF1) or von Recklinghausen syndrome is an autosomal dominant disorder that affects the multisystem in the body with complex presentation caused by the neurofibromin gene mutation on chromosome 17. These patients tend to develop soft tissue sarcomas more than the general population. Leiomyosarcoma is a malignant soft tissue tumor that may occur in patients with NF1 in rare cases. We present a case of a rare development of leiomyosarcoma in a 45-year-old female patient with a history of NF1. She developed a progressively growing mass in the left axilla associated with numerous neurofibromas and axillary freckling. MRI revealed a heterogeneous large mixed signal intensity mass in the left axilla, and the diagnosis was confirmed through biopsy.

## Introduction

Neurofibromatosis type 1 (NF1) is an autosomal dominant disorder affecting people across the world with an incidence of 1/2500 to 1/3000 [1]. NF1 is a diverse disease presenting clinically with various manifestations. Apart from affecting an individual cosmetically, in certain cases, a life-threatening and grave presentation may be observed. Moreover, the possibility to foresee the development of any particular complication in an individual is often diagnostically challenging [2]. NF1 has been associated with the occurrence of soft tissue tumors presenting aggressively with undesirable outcomes in most cases [3]. In comparison with the general population, patients with NF1 are more prone to developing malignancies by four to six times, and the occurrence of soft tissue tumors reaches up to 34 times as compared to an unaffected individual [4]. Leiomyosarcoma is a type of soft tissue tumor growing in smooth muscles with an aggressive nature. NF1 had been rarely presented concurrently with leiomyosarcoma of the bone, an intracranial leiomyosarcoma, or a leiomyosarcoma in the thigh. However, to the best of our knowledge, leiomyosarcoma in a patient with neurofibromatosis has not yet been reported in the axilla. Here, we present a case of axillary leiomyosarcoma in a 45-year-old female patient with NF1.

## Case presentation

**Figure 1:**
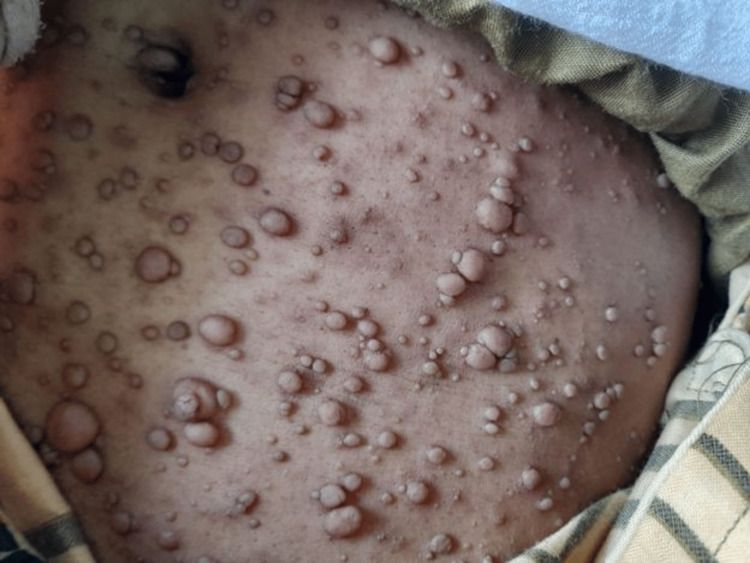
Neurofibromas in NF1

A 45-year-old female with NF1 presented with a growing mass associated with mild pain in the left axilla for two months. The mass gradually increased in size during this time with increasing pain and discomfort in the movement of the left upper extremity. On physical examination, she had a subcutaneous soft mass about 6x5 cm in size with ill-defined margins in the left axilla which was mobile, non-tender, and non-translucent. The left upper extremity range of motion was normal but had mild discomfort with movement. She had numerous neurofibromas all over the body and axillary freckling (Figure 1).

**Figure 2:**
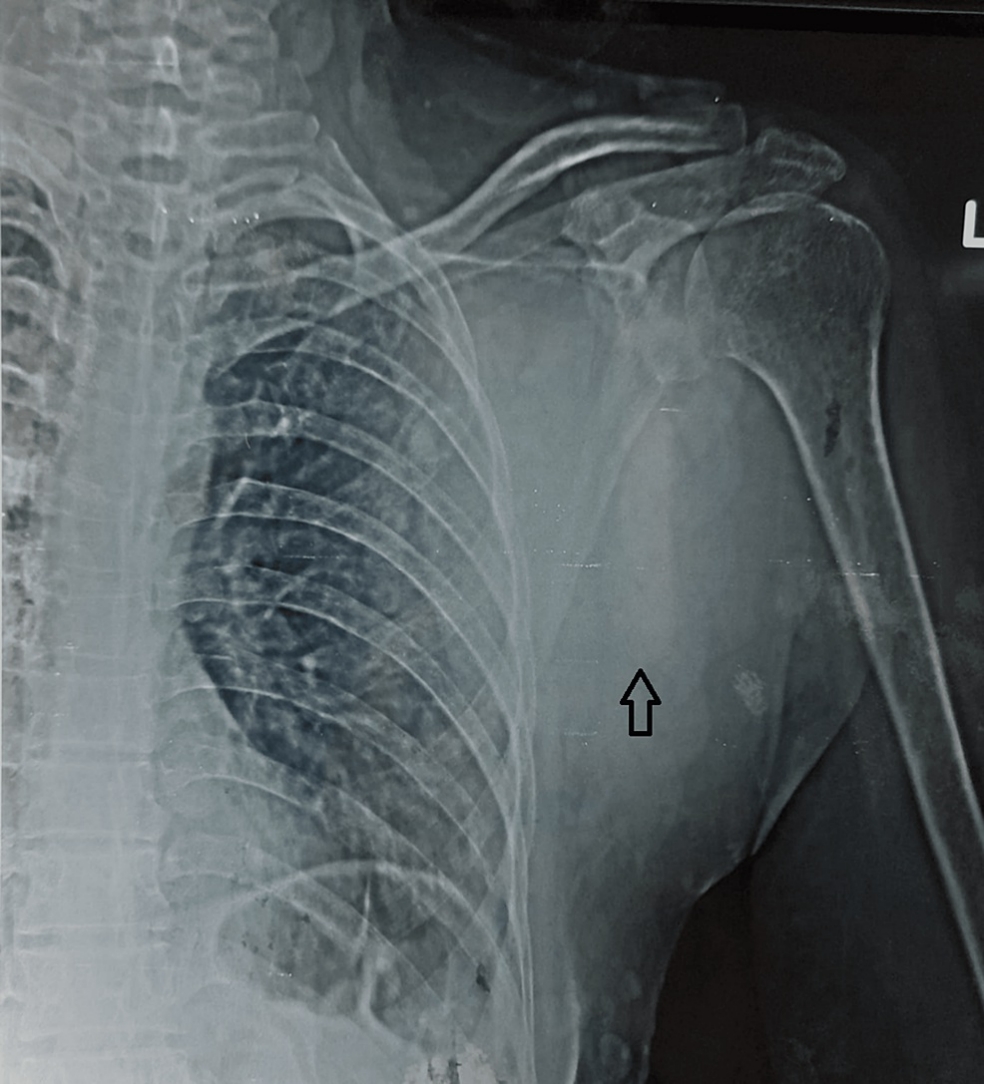
X-ray chest showing large lobulated soft tissue seen within the subcutaneous tissue along the left lateral scapular margins

Lab investigations disclosed that her hemoglobin was 12.9 g/dL, white blood cell count was 14,200/cm3, and platelet count was 4,29,000/cm3. Her basic metabolic panel was essentially normal. X-ray chest showed large lobulated soft tissue seen within the subcutaneous tissue along the left lateral scapular margins. No sclerosis, cortical defect, or calcification was seen in the underlying scapula and humerus (Figure 2).

**Figure 3:**
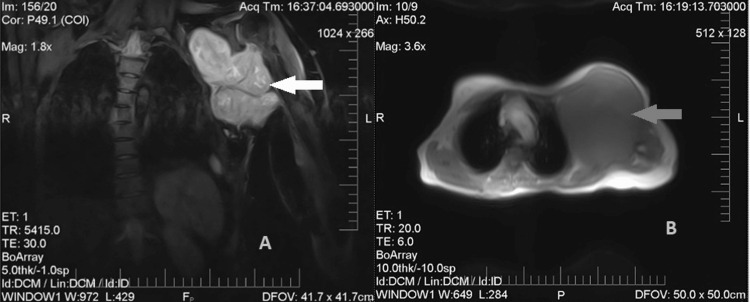
MRI chest coronal (A) and axial (B) views showing a heterogeneous large mixed signal intensity mass in the left axilla shown by white arrowhead in image A and gray arrowhead in image B

MRI of the left shoulder joint showed a 14.5x13.3x11.8 cm heterogeneous large mixed signal intensity mass in the left axilla. The lesion indents into the left upper anterior intercostal spaces and also abuts the left proximal humerus with no frank bony infiltration. The lesion is in line with the left coracobrachialis muscle, also stretching the overlying pectoralis major and adjacent latissimus dorsi muscles (Figure 3).

A Tru-cut biopsy revealed multiple cores of neoplastic lesion composed of pleomorphic round to elongated atypical cells having eosinophilic cytoplasm and atypical hyperchromatic nuclei with inconspicuous nucleoli. There was a high level of mitotic activity, with 23 mitotic figures observed in every ten high-power fields, and over 50% of the area showed necrosis. The biopsy report classified the tumor as FNCLCC (Fédération Nationale des Centres de Lutte Contre Le Cancer) grade 3 sarcoma with patchy weak positive anti-smooth muscle antibody and H-caldesmon on immunohistochemical analysis, which are in favor of leiomyosarcoma. Moreover, other markers such as desmin, S100, CD117, and DOG1 (discovered on gastrointestinal stromal tumors protein 1) were found to be negative. She refused any possible surgical resection of the tumor and was referred to the oncology department for possible anthracycline-based chemotherapy but was subsequently lost to follow-up.

## Discussion

NF1, an autosomal dominant disorder, is mainly diagnosed by the presence of benign neurofibromas, café au lait spots, lisch nodules of the iris, and the presence of learning disabilities and skeletal abnormalities in some cases patients [5]. The NF1 gene encodes a tumor suppressor protein neurofibromin which is a negative regulator of the RAS/MAP kinase pathway, with most patients having both a normal copy and a mutated one. The second somatic hit according to Knudson's two-hit hypothesis renders the normal NF1 gene ineffective resulting in the complete elimination of functional neurofibromin and the formation of benign neurofibromas [1]. There may be additional mutations at different genetic loci with a complex multistep process in the formation of neurogenic and non-neurogenic sarcomas [6]. NF1 patients may have an increased possibility of the occurrence of malignant neoplasm which may be linked to the tumor suppression effect of the NF1 gene [7]. The transformation of benign neurofibromas into aggressive forms, known as malignant peripheral nerve sheath tumors (MPNST), in 8% to 13% of patients can occur for which certain biomarkers had been identified for early detection in NF1 patients susceptible to MPNST such as insulin-like growth factor binding protein 1 (ILGBP1) and regulated upon activation, normal T cell expressed and secreted (RANTES) but in unprecedented cases, malignant tumors not relating to the nervous system may occur such as leiomyosarcoma [8,9].

Leiomyosarcoma had rarely been reported in patients suffering from neurofibromatosis with cases reported in the hand, thigh, bone, intracranial pelvis, colon, and bladder with most of them being reported in adolescents. Izzeddin et al. reported a case of primary bone leiomyosarcoma in a 14-year-old kid with plexiform neurofibroma [10]. Similarly, a rare association between ileal leiomyosarcoma, NF1, and gastroparesis was reported in Italy, and a case of intracranial leiomyosarcoma was reported in Canada [11,12]. Interestingly, our case is one such rare concurrence of leiomyosarcoma and NF1 in a female in her 40s, which makes it unique from the other cases. No such biomarkers are known for early recognition of susceptible NF1 patients in cases of such tumors.

The development of such tumors in NF1 patients needs awareness and attention in dealing with patients with NF1 for early diagnosis and timely management of such complications for better outcomes of patients.

## Conclusions

NF1 with leiomyosarcoma has rarely been reported in the medical literature. This case can be added to the few reported cases of leiomyosarcoma in a patient with neurofibromatosis. Such cases highlight the possibility of malignant tumors in NF1 patients besides MPNST. The case highlights the importance of surveillance and investigating patients comprehensively for the early diagnosis of such malignant tumors in NF1 for a more satisfactory outcome and timely management.
